# The Application of Protein Concentrate Obtained from Green Leaf Biomass in Structuring Nanofibers for Delivery of Vitamin B12

**DOI:** 10.3390/foods13101576

**Published:** 2024-05-18

**Authors:** Bojana Balanč, Ana Salević-Jelić, Verica Đorđević, Branko Bugarski, Viktor Nedović, Predrag Petrović, Zorica Knežević-Jugović

**Affiliations:** 1 Innovation Centre of Faculty of Technology and Metallurgy, University of Belgrade, Karnegijeva 4, 11000 Belgrade, Serbia; bisailovic@tmf.bg.ac.rs (B.B.); ppetrovic@tmf.bg.ac.rs (P.P.); 2 Faculty of Agriculture, University of Belgrade, Nemanjina 6, 11080 Beograd, Serbia; ana.salevic@agrif.bg.ac.rs (A.S.-J.); viktor.nedovic@mpn.gov.rs (V.N.); 3 Faculty of Technology and Metallurgy, University of Belgrade, Karnegijeva 4, 11000 Belgrade, Serbia; branko@tmf.bg.ac.rs (B.B.); zknez@tmf.bg.ac.rs (Z.K.-J.)

**Keywords:** nanofibers, electrospinning, pumpkin leaves protein, gelatin, vitamin B12

## Abstract

Nanofibers made of natural proteins have caught the increasing attention of food scientists because of their edibility, renewability, and possibility for various applications. The objective of this study was to prepare nanofibers based on pumpkin leaf protein concentrate (LPC) as a by-product from some crops and gelatin as carriers for vitamin B12 using the electrospinning technique. The starting mixtures were analyzed in terms of viscosity, density, surface tension, and electrical conductivity. Scanning electron micrographs of the obtained nanofibers showed a slight increase in fiber average diameter with the addition of LPC and vitamin B12 (~81 nm to 109 nm). Fourier transform infrared spectroscopy verified the physical blending of gelatin and LPC without phase separation. Thermal analysis showed the fibers had good thermal stability up to 220 °C, highlighting their potential for food applications, regardless of the thermal processing. Additionally, the newly developed fibers have good storage stability, as detected by low water activity values ranging from 0.336 to 0.376. Finally, the release study illustrates the promising sustained release of vitamin B12 from gelatin-LPC nanofibers, mainly governed by the Fickian diffusion mechanism. The obtained results implied the potential of these nanofibers in the development of functional food products with improved nutritional profiles.

## 1. Introduction

Nanofibers made of natural proteins have caught the increasing attention of food scientists in recent years because of their edibility, renewability, availability, and possibility for various applications, such as bioactive encapsulation, enzyme immobilization, food coating, food texture modification, the production of active packaging systems, or even wider [1,2,3]. Generally, the use of nanofibers allows for a large surface area, high porosity, and improved encapsulation efficiency. Electrospinning is an electro-hydrodynamic, simple, and cost-effective technique used for the fabrication of nanofibers using different raw materials [4,5]. In the context of food-grade applications, it is employed to create structures that can encapsulate and protect sensitive bioactive compounds, such as vitamins. There has been interest in nanofibers made of mixed biopolymers [6]. A prerequisite for obtaining nanofibers by electrospinning is to use at least one spinnable biopolymer. Gelatin is one of the few highly spinnable proteins with many favorable properties, such as biodegradability, biocompatibility, and low cost [7,8]. However, sole gelatin nanofibers do not exert bioactivity [7]. Therefore, gelatin was successfully blended with some globular proteins that cannot be spun by themselves [9]. In this way, the extra nutritional value of gelatin-based nanofibers was achieved by using whey protein isolate, ovalbumin, soy protein isolate, or sodium caseinate. In addition, protein nanofibers may interact with natural polyphenols present in protein isolates, which results in improved stability [2].

The need for cheap and high-quality protein is growing. Leaves available as by-products from some crops on a large scale could potentially be used as a major protein source for food applications. Most of the studies on leaf protein in the past decades focused on developing extraction protocols for soluble protein recovery in the form of leaf protein concentrate (LPC) [10,11], while its utilization as an ingredient in human food is still at the early stages [12]. Rubisco, the main compound of the soluble protein fraction, is found to have interesting functional properties (high foaming capacity, good solubility at food pH, and ability to form gels at low concentrations and low temperatures) [13,14]. However, the potential of this protein to be processed into a structurally appealing product has not been explored yet. To our knowledge, the functionality of rubisco when incorporated into fibrous mats has not yet been studied. This study aimed to investigate the spinnability of a mixed solution of gelatin and leafy vegetable protein concerning changes in the physicochemical properties of solutions. Pumpkin (*Cucurbita pepo*) will be used as a source of leaf protein. This annual green leafy vegetable is widely cultivated for its fruits and seeds, while leaves are mainly underutilized despite huge potential; only in the southern part of Nigeria do the leaves of pumpkin, *Telfairia occidentalis*, constitute an important vegetable with a protein content of ~30% dry matter [15,16]. According to Tanimowo Fadupin et al., the crude protein content in the leaf of *Cucurbita pepo* is 7.3% wet matter [17].

Electrospun gelatin nanofibers instantly dissolve in cold water and can be used for designing fast-dissolving systems aimed at supplementing food with nutraceuticals [8,18,19]. Higher stability of sensitive compounds was achieved by their incorporation in gelatin nanofibrous films [18,20]. In this study, we test the hypothesis that gelatin-leaf protein can serve as a carrier for vitamin B12. Vitamin B12, or cobalamin, is an essential vitamin present in milk, eggs, meat, and shellfish. It exhibits important biological functions such as red blood cell formation, DNA synthesis and regulation, cell division, and neurological functions [21,22]. However, it becomes degraded and loses its activity upon heating the food and during storage processes [23]. Thus, vitamin B deficiency becomes an increasing health issue, especially among the elderly population, vegetarians, and pregnant women [24,25]. Therefore, different structures with encapsulated vitamins have been developed in the last couple of years [26,27,28]. High hydrophilicity, high molecular weight (1355.38 g/mol), and sterically hindering of this compound may cause difficulties for encapsulation. The novel herein-developed protein matrices containing vitamin B12 may exhibit high potential to contribute to the food industry.

## 2. Materials and Methods

### 2.1. Materials

The pumpkin leaves used for protein extraction were collected on fields owned by the company JS&O (Novo Milosevo, Serbia). Beef-skin gelatin (Type B, Bloom 220) was purchased from Gelnex (Santa Catarina, Brazil), and Cyanocobalamin (vitamin B12) was provided from Sigma Chemical Co. (St. Louis, MO, USA). Glacial acetic acid (>99%) was purchased from Macron Fine Chemicals (Centre Valley, PA, USA). All other chemicals were of analytical grade, and the water used was double-distilled.

### 2.2. Preparation of Pumpkin Leaf Protein Concentrate (LPC)

Briefly, about 300 g of fresh pumpkin leaves were mechanically processed so that a protein-rich juice was squeezed out of the leaves. Then, the heat coagulation step was applied for the precipitation of green proteins from the obtained green juice (at 55 °C, 30 min). The final step implied the addition of acid to so-called “brown juice” to change the solubility of the proteins and provide their precipitation. Thus, the pH was adjusted to 4.5 using 2 M HCl, as the isoelectric point of Rubisco is between 4.4 and 4.7 [29]. After the centrifugation, the white protein fraction was separated and further subjected to the freeze-drying process. In this way, about 1 g of LPC was prepared.

### 2.3. Preparation of the Solutions

Gelatin (20% *w/v*) was dissolved in acetic acid (30% *v/v*) at room temperature for 24 h to ensure complete hydration. Then, the LPC was added to the gelatin solution at a concentration of 10 mg/mL, and again, the mixture was stirred overnight at room temperature. A vitamin B12 solution was added directly 30 min before the electrospinning procedure at a final concentration of 0.4 mg/mL. For viscosity, surface tension, density, and conductivity measurements, vitamin B12 was dissolved in acetic acid (30% *v/v*) to obtain the same final concentration. 

### 2.4. Characterization of the Gelatin-LPC Solutions

Previously prepared solutions were characterized in terms of electrical conductivity using a digital Benchtop Conductivity Meter (HI 2315, HANNA Instruments, Ltd., Temse, Belgium).

The surface tension was measured using a Krüss K20 tensiometer (Krüss GmbH, Hamburg, Germany). The Wilhelmy plate method was applied for those measurements. A cleaned platinum plate was dipped into the examined solutions. The tensiometer detects the force that is needed to pull the plate out of the sample. This maximum value of the force is detected just before the plate leaves the solution, and this value is proportionate to the surface tension.

The density was determined using the same instrument equipped with the DE01 set.

The viscosity of the solutions was measured on an IKA ROTAVISC LO-VI viscometer (KA, Staufen, Germany) using spindle SP1. 

All measurements were carried out in triplicate at room temperature (25 ± 1 °C). 

### 2.5. Electrospinning Processing

**Figure 1:**
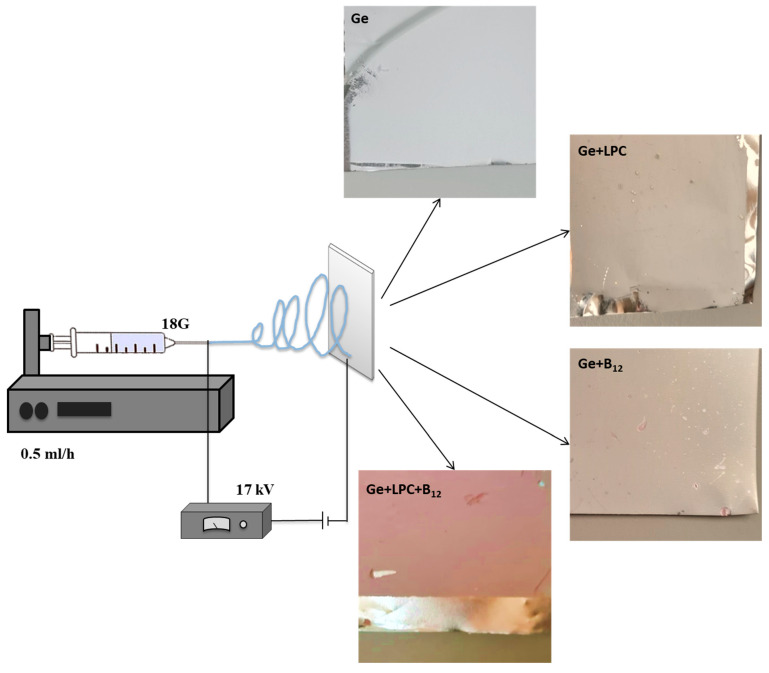
Schematic illustration of electrospinning set used in the study.

Gelatin solutions (plain, with LPC, or with LPC and vitamin B12) were electrospun through a blunt stainless-steel needle (18 G) at a steady flow rate of 0.5 mL/h using a syringe pump (Razel Scientific Instruments, Stamford, CO, USA). An electric field (17 kV) was applied between the positively charged needle and the grounded metallic collector plate. The distance between the needle tip and the collector plate was 10 cm (Figure 1). The process occurred at ambient temperature (~25 °C).

### 2.6. Nanofibers Morphology and Size

The surface morphology of nanofibers was determined by scanning electron microscopy (SEM, model TESCAN MIRA3XMU, Brno, Czech Republic) using a voltage of 10 kV and a magnification of 50,000. The size distribution of the obtained nanofibers was determined from the SEM images using the Image J software V 1.8.0, which analyzed no less than 100 fibers. 

### 2.7. Encapsulation Efficiency

The encapsulation efficiency of vitamin B12 into gelatin-based nanofibers was valued as described by Coelho et al. [26]. Namely, vitamin B12 released instantly at time zero corresponds to the vitamin outside of the fibers. Using this data, it is possible to calculate the encapsulation efficiency as the ratio between the amount of B12 in gelatin structures and the total amount of B12.

### 2.8. Fourier Transform Infrared Spectroscopy

Fourier transform infrared (FT-IR) spectroscopy was employed to evaluate the structural properties of the fibrous mats and the chemical interactions between their constituents. The as-prepared samples were subjected to analysis in attenuated total reflection mode (ATR) in the wavenumber range of 4000–600 cm^−1^ with a resolution of 4 cm^−1^ and 100 accumulations per scan using an IRAffinitty-1S (Shimadzu, Kyoto, Japan). 

### 2.9. Thermogravimetric Analysis 

Thermogravimetric analysis (TGA) was employed to show the mass losses of the samples upon heating from ambient temperature (30 ± 2 °C) to 250 °C. Keeping in mind that thermal treatments of foods in processing and preparation often involve temperatures up to 250 °C, this analysis projected the stability of the samples upon heating. The experiments were performed using the TG/DTA SetSys 2400 instrument (Setaram, Cailure, France). The heating rate was 10 °C/min and airflow was maintained at 20 mL/min.

### 2.10. Differential Scanning Calorimetry (DSC)

The thermal properties of the nanofibers were examined by the DSC technique. The samples were placed in DSC aluminum pans, which were hermetically sealed and analyzed using a DSC131 Evo (SETARAM Instrumentation, Caluire, France). The pens with samples were heated from 30 to 250 °C with a constant heating rate of 10 °C/min under a nitrogen atmosphere at a flow rate of 20 mL/min.

### 2.11. Water Activity

The water activity (Aw) values of the samples were measured using a water activity measurement device (LabSwift-aw, Novasina AG, Lachen, Switzerland).

### 2.12. Release Study

The release study was conducted by monitoring the vitamin B12 release directly into the water (pH 7), and the amount of released vitamin B12 was determined by measuring the absorbance (at 350 nm) up to the moment where the maximum occurred and stabilized. About 1 g of fiber was immersed in 20 mL of the medium at 25 °C under shaking at 300 rpm. Samples were taken from the release medium at predetermined time intervals. The release of vitamin B12 was analyzed using a microplate spectrophotometer (Multiskan^TM^ GO, Thermo Scientific^TM^, Waltham, MA, USA). The volume was kept constant by adding a fresh medium. The release measurements were carried out in triplicate.

The mechanisms governing the release of vitamin B12 from the as-prepared structures were studied using a few kinetic models, such as Higuchi (Equation (1)), Ritger–Peppas (Equation (2)), Kopcha (Equation (3)), and Peppas–Sahlin (Equation (4)):

m_t_/m_∞_ = k·t^1/2^ (1)

m_t_/m_∞_ = k·t^n^ (2)

m_t_/m_∞_ = A·t^0.5^ + B·t (3)

m_t_/m_∞_ = k_1_·t^m^ + k_2_·t^2m^ (4)

where m_t_ and m_∞_ are the amounts of released vitamin B12 at time t and at infinite time, while k, and t correspond to the release constant and release time, respectively. Exponent n determines the mechanism of vitamin B12 release from the carrier: 0.45 < n < 0.89 defines non–Fickian diffusion, *n* ≥ 0.89 defines erosion mechanism, while n ≤ 0.45 defines Fickian diffusion. Index m is the diffusion exponent. The parameters k_1_ and A are Fickian constants, and the values k_2_ and B are the erosion constants [19,30].

### 2.13. Statistical Analysis

The results were statistically analyzed using IBM SPSS Statistics 25 software (Armonk, New York, NY, USA). Depending on the homogeneity of variance test, significant differences between the samples were determined by one-way analysis of variance (ANOVA) with Tukey post hoc or Kruskal–Wallis H test with Mann–Whitney U test. A significance level of 0.05 was set.

## 3. Results and Discussion

In the electrospinning process, the competition among conductivity, tension force, and viscosity in a limited range affects fiber morphology and diameters. In this study, gelatin was spun from the aqueous acetic acid solution (30%, *v/v*) at a concentration of 20% (*w/v*). According to the literature [31,32], a 20–40% (*w/v*) concentration range of gelatin provides regular nanofiber formations under wide spectra of electrospinning process conditions (applied voltage and feed rate), while below a certain concentration, the particles will develop instead of fibers. In our experiments, the electrospinning of a sole LPC aqueous solution was not an effective process because the protein molecules in globular conformation do not display the necessary entanglements or interchain connections to electrospin. 

**Figure 2:**
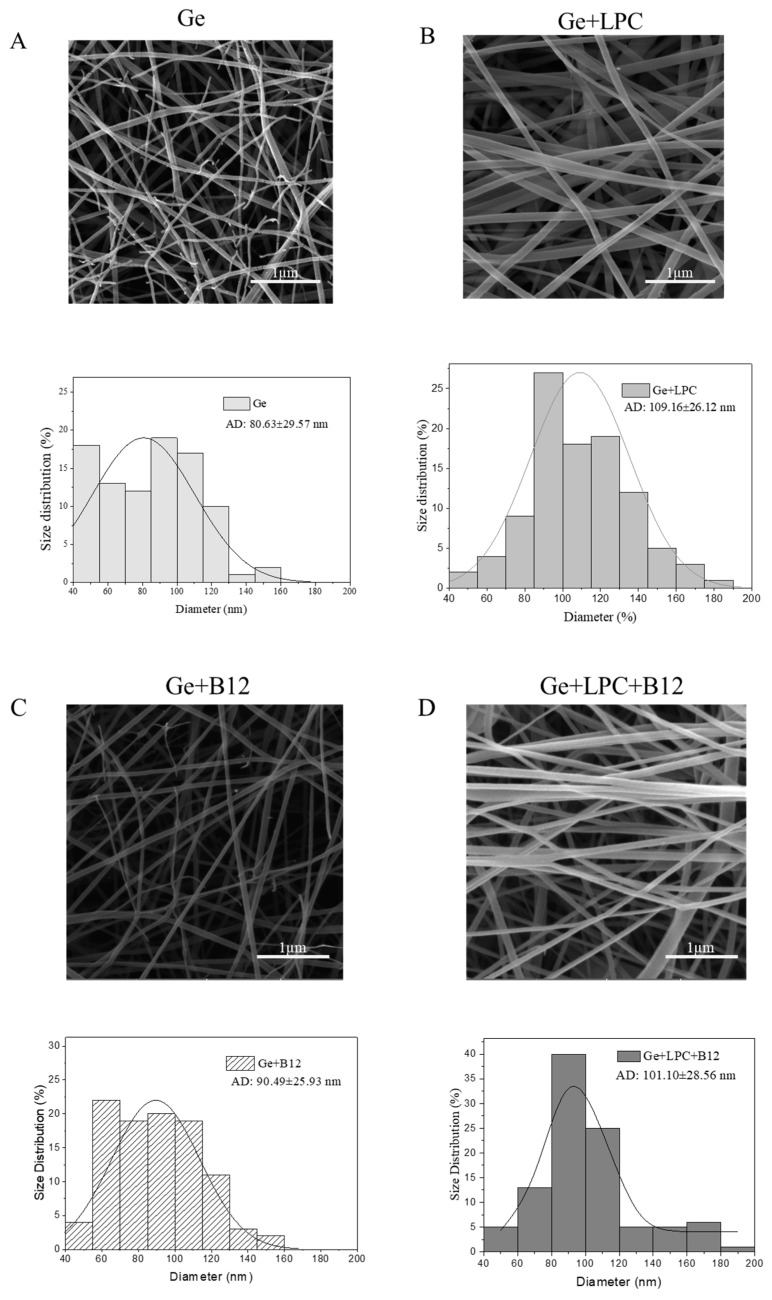
SEM images and fiber diameter distributions of (**A**) gelatin nanofibers (Ge), (**B**) gelatin nanofibers with LPC (Ge+LPC), (**C**) gelatin nanofibers with vitamin B12 (Ge+B12) and (**D**) gelatin nanofibers with LPC and vitamin B12 (Ge+LPC+B12). Data are expressed as mean ± SD (*n* = 100).

Electrospinning under ambient conditions appeared to be a promising technique for encapsulating vitamin B12 with high encapsulation efficiency. The vitamin encapsulation efficiency (EE) of 97 and 99% was determined (for Ge+B12 and Ge+LPC+B12, respectively), which is in agreement with the result of Coelho et al., who used zein to encapsulate this vitamin by electrospinning and reported values between 61 and 100% depending on zein and vitamin concentrations and process parameters [26]. The electrospun formulations were evaluated by SEM to scrutinize their morphology and size. Ultra-thin nanofibers (81–109 nm) with homogeneous formats were developed, as shown in Figure 2. The use of green leaf protein corresponded to the formation of smoother and continuous fibers compared to somewhat choppy, branched fibers of solely gelatin (Figure 2B vs. Figure 2A). The formation of branched fibers in the case of sole gelatin indicates instability of the jet during electrospinning, which can split it into smaller jets or eject smaller jets, reducing local charge per surface area [33]. The absence of branching and more regular fibers’ morphology induced by the LPC and vitamin addition could be due to the increased conductivity and electrical forces stabilizing the jet against splitting. This suggests improved molecular entanglements through the interactions between both constituents. When considering industrial applications, the size and polydispersity of the shapes are important factors to ensure the replicability and consistency of the products. LPC was more homogenous, filamentous, and thicker compared to pure gelatin. No obvious effect of vitamin B12 encapsulation on fiber morphology was observed. 

The morphological results mainly corresponded to the properties of the blend solutions shown in Table 1. Pure gelatin solution (20%, *w/v*) had a high electrical conductivity of 4.10 mS/cm, and the value increased upon adding LPC since the LPC solution also expressed electrical conductivity. The addition of vitamin B12 further increased the electrical conductivity of the solution. This can be related to the decrease in viscosity, as it was reported that the decreased viscosity might affect the mobility of the charged species in the solutions, increasing conductivity [34]. This explains our morphological observations (Figure 2), proving that higher charge density generally generates smoother fibers because of the more intense whipping instability of the liquid jet. The surface tension of the polymer solution also plays a significant role in electrospinning. Leaf protein decreased the surface tension of the initial gelatin solution, which meant that viscoelastic forces easily overcame the surface tension, resulting in smoother fibers. The viscoelastic property of a feed solution is the most important parameter during the electrospinning process. However, measuring the viscoelastic properties is quite impractical, and instead, the viscosity of the solution could provide a solid indication of the spinnability and size of the electrospun fibers [31,32]. In general, the greater solution viscosity provides ticker fibers. A slight increase in viscosity was determined upon the addition of the leaf extract to the gelatin solution, which further resulted in thicker fibers.

### 3.1. Fourier Transform Infrared Spectroscopy

**Figure 3:**
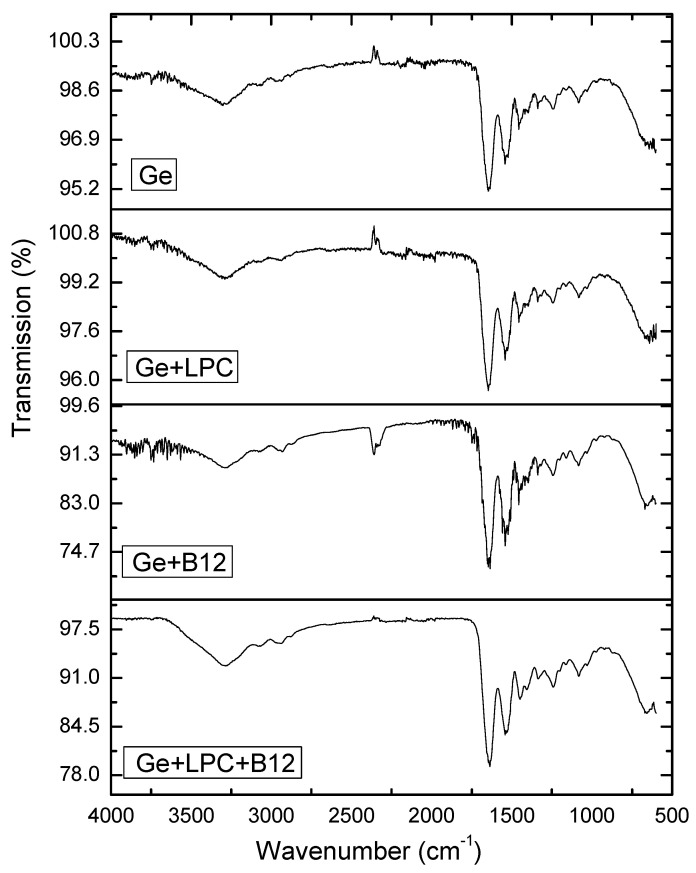
Infrared spectra of different electrospun nanofibers.

Figure 3 shows the spectra of gelatin-based nanofibers. The neat gelatin nanofibers showed characteristic bands in agreement with those previously identified in the literature for gelatin-based materials [35,36]. These bands were as follows: 3294 (hydrogen bonding and N-H stretching, amide A), 2936 (C-H stretching vibrations of aliphatic groups), 1647 (C=O stretching vibrations, amide I), 1541 (C-N bending and stretching vibrations, amide II), 1456 (N–H bend and C-N stretching combination band, NH3^+^ symmetric deformation), 1339 (C–H deformation of methyl group), 1238 (C-N stretching and N-H bending, amide III), and 1082 (C-O stretching) cm^−1^.

The nanofibers obtained by blending gelatin with pumpkin leaf extract showed a similar FT-IR spectrum as that previously discussed for neat gelatin nanofibers. The blending did not induce new bands nor shifts of gelatin-originating bands, which implies efficient, physical blending of gelatin and pumpkin leaf extract without phase separation or chemical interactions. The spectra obtained after vitamin B12 encapsulation, within the fibers based on the sole gelatin and blend of gelatin and pumpkin leaf extract were similar to those of the neat matrix. No new bands or band splits were observed after vitamin B12 encapsulation suggesting efficient vitamin B12 encapsulation within sole gelatin fibers and gelatin-pumpkin leaf extract blend-based fibers. Of note, vitamin B12 encapsulation shifted the band described to amide I. This band was shifted from 1647 cm^−1^ for the neat gelatin and gelatin-pumpkin leaf extract blend fibers to 1635 and 1636 cm^−1^ after vitamin B12 encapsulation within sole gelatin fibers and gelatin-pumpkin leaf extract blend-based fibers, respectively. This spectral change implies intermolecular interactions between the protein-based matrix and the active compound and changes in proteins’ structure induced by the active compound incorporation [36]. According to the literature, cobalamin interacts with proteins with different binding affinity for different proteins by hydrogen bond, hydrophobic force, and π-π interactions [37]. 

### 3.2. Thermal Stability Analysis

**Figure 4:**
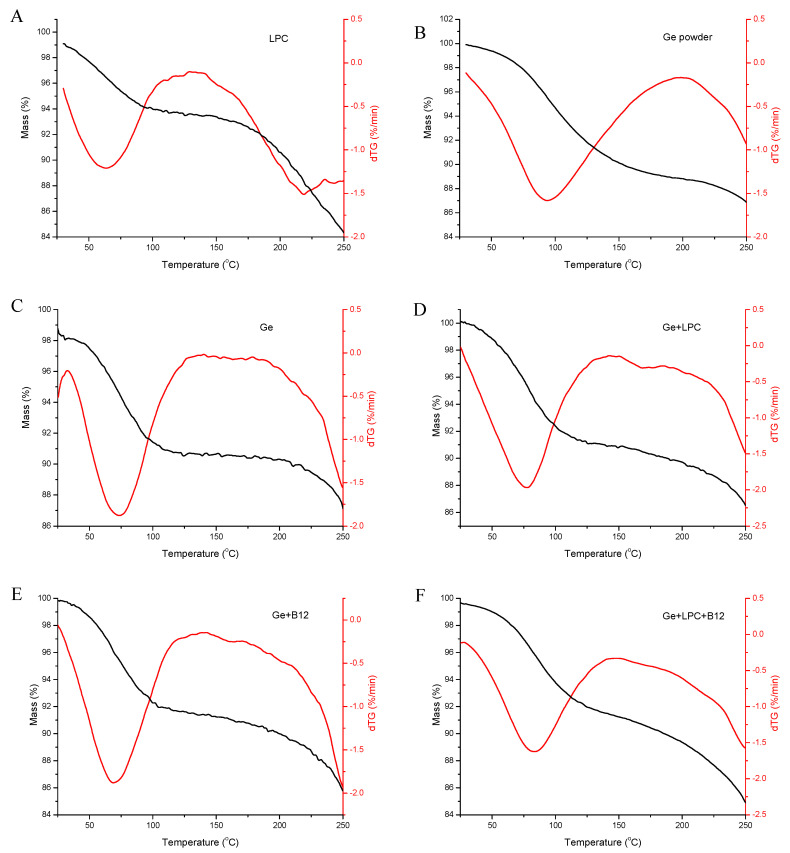
Thermogram and their first derivatives of (**A**) raw leaf protein concentrate (LPC), (**B**) gelatin powder (Ge powder), (**C**) gelatin nanofibers (Ge), (**D**) gelatin nanofibers with LPC (Ge+LPC), (**E**) gelatin nanofibers with vitamin B12 (Ge+B12), and (**F**) gelatin nanofibers with LPC and vitamin B12 (Ge+LPC+B12).

Figure 4 presents the weight loss of LPC, gelatin powder, and the obtained fibers. The temperature range was chosen according to the intended final usage as a food ingredient. Keeping in mind that food is usually baked up to 250 °C, the weight loss was monitored up to this temperature. The first stage of weight loss for the fibers was starting from room temperature up to around 75 °C. This can be attributed to the bulk moisture of nanofibers and, thus, to the evaporation of free and weakly bonded water. The mass reduction was about 6%. The second weight loss started around 220 °C and went further with the temperature increase. This reduction was presumably related to protein degradation [38] and was also confirmed by DSC results. LPC exhibited degradation at lower temperatures compared to raw gelatine. The addition of vitamin B12 did not affect the thermal stability of the fibers; namely, cyanocobalamin is thermally stable in the investigated temperature range [39,40] Obviously, the prepared fibers have good thermal stability up to 220 °C, highlighting their potential for food applications, regardless of the thermal processing.

### 3.3. DSC

**Figure 5:**
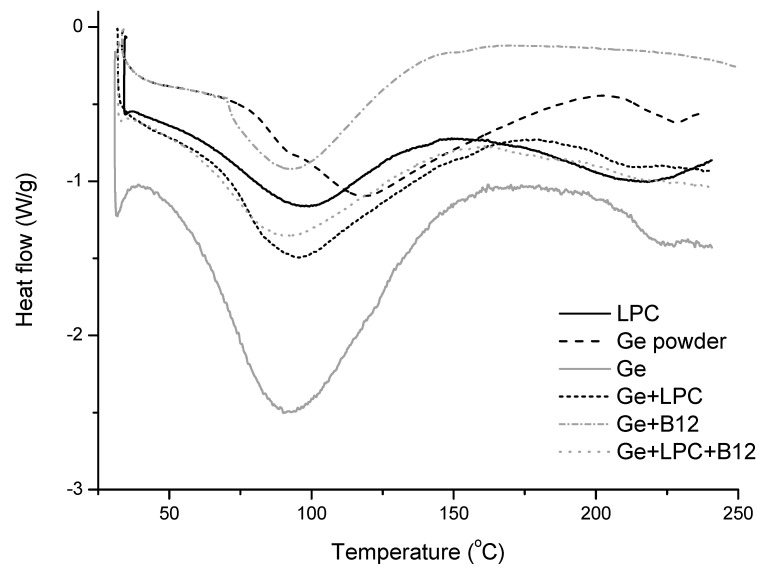
DSC curves of raw leaf protein concentrate (LPC), gelatin powder (Ge powder), gelatin nanofibers (Ge), gelatin nanofibers with LPC (Ge+LPC), gelatin nanofibers with vitamin B12 (Ge+B12) and gelatin nanofibers with LPC and vitamin B12 (Ge+LPC+B12).

The thermal behavior was also studied by DSC analysis; thus, the effects of the addition of LPC and vitamin B12 encapsulation in nanofibers are presented in Figure 5 and summarized in Table 2. The thermogram of the unloaded LPC showed two endothermic peaks, the first around 96 °C with an enthalpy of 161.65 J/g, and the second around 220 °C. The first peak is presumably formed due to denaturation, while the second is related to the decomposition of LPC [7]. In the literature, it can be found that denaturation peaks for protein isolates vary between 69 °C and 109 °C for *Spirullina* protein isolates [41] and between 87 and 107 °C for legume protein isolates [42]. Namely, it is known that the thermal characteristics of globular proteins may be related to their thermal-induced aggregation and gelation [42,43,44]. As the denaturation temperature increases, the thermal stability of proteins increases, and the polypeptides have a more compact tertiary structure [42]. The DSC curve of gelatin powder shows the appearance of peaks at 118 °C and 228 °C. The first transition can be assigned to the devitrification of α-amino acid blocks, whereas the second one can be assigned to the devitrification of the blocks of amino acids (hydroxyproline and proline) [45]. Compared to gelatin powder, all the thermograms of fibers showed the first glass transition peak shifted to lower temperatures (90–95 °C) with higher values of enthalpy. The second peak is much weaker and also shifted to somewhat lower temperatures (211–223 °C). This occurrence can be explained by the electrospinning process, by which the segmental mobility of fibrous polymers is increased [8]. In addition, other authors also reported a decrease in the Tg of nanofibers due to interactions between the fiber constituents [46].

### 3.4. Water Activity (Aw)

Water activity points to the available free water in the examined sample; thus, the parameter is important to define the microbial stability of the sample. For example, at optimum pH and temperature, the minimal Aw values required for the growth of pathogenic bacteria are 0.85–0.86. On the other hand, yeasts and molds tolerate lower Aw values, but usually not below 0.62 [47]. The results for the prepared fibers are shown in Table 3. The values obtained for native gelatin powder were similar to those reported by Rather et al. and also similar to the Aw values of other samples [48]. The Aw values of the prepared fibers ranged from 0.336 to 0.376, and there were no statistically significant differences among the samples, suggesting that the addition of protein or vitamin B12 did not affect the Aw values of gelatin fibers. The obtained Aw values of the developed fibers were lower than those of zein-based electrospun fibers incorporating gallic acid [49]. Accordingly, the newly developed fibers have good storage stability.

### 3.5. Release Study

**Figure 6:**
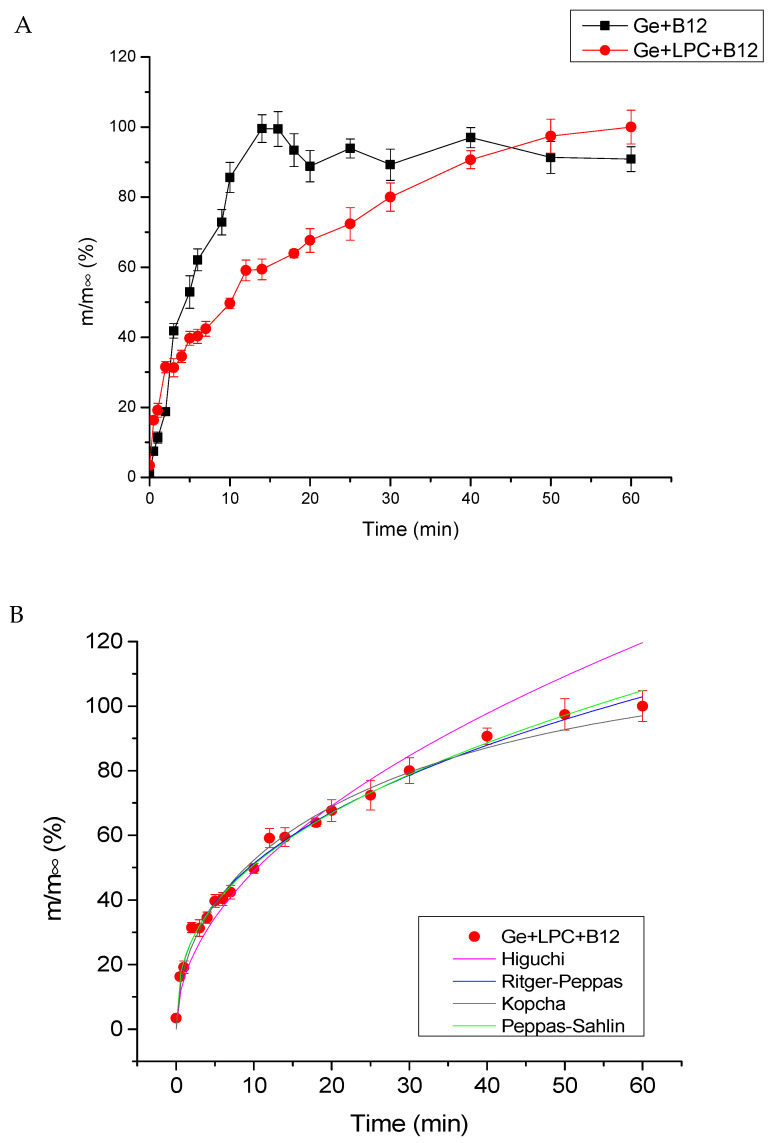
(**A**) Profiles of vitamin B12 release from gelatin and gelatin fortified with LPC nanofibers (each data represents the average value of three replicates). (**B**) Kinetics fitting of vitamin B12 release from the gelatin fortified with LPC nanofibers, fitting data are presented by lines.

The effect of the protein matrices on vitamin B12 release behavior was assessed. The release profile of Ge+B12 vs. Ge+LPC+B12 is depicted in Figure 6A. It is evident that the release of vitamin B12 from fibers fortified with LPC is significantly slower. For the purpose of comparison, after 15 min, vitamin B12 was almost completely released from solely gelatin fibers (>98%), while for the same period, only 60% of the vitamin was released from fibers with LPC. Due to high specific surface area, amorphous structure, and high porosity, neat gelatin nanofibers rapidly dissolve in water (unlike bulk gelatin) by forming a white gel [8,19,50]. However, the potential drug-polymer interactions may slow down matrix erosion and drug release by different mechanisms (shielding of matrix terminal residues, decreasing the porosity of the matrix, decreasing the drug partition to the water-filled micropores). Also, the addition of LPC increased the number of functional groups available for interactions.

The kinetic fitting of vitamin B12 released from the fibers obtained in this study was also performed. Four different models (Kopcha, Peppas–Sahlin, Ritger–Peppas, and Higuchi) were applied, and the results are presented in Figure 6B and Table 4. The Ritger–Peppas equation was found to be the most suitable one to describe the release mechanism of vitamin B12 from Ge+LPC+B12 nanofibers. The main criteria to calculate the goodness of fit are the values of the coefficient of determination (R2) and The Root Mean Square Error (RMSE). Generally, as the value of R2 is closer to 1, the model perfectly predicts values. On the other hand, as the RMSE value is lower than 10, the model fits the data superbly. For our sample, fitting parameter n is lower than 0.45 (Ritger–Peppas), indicating Fickian diffusion as a mechanism of vitamin B12 release. To confirm this, constant k_1_ is higher than k_2_, indicating the main mechanism governing the release is the same: Fickian diffusion (by Peppas–Sahlin).

## 4. Conclusions

Food-grade nanofibers based on pumpkin leaf protein and gelatin for the delivery of vitamin B12 were fabricated using the electrospinning process, and it was an innovative approach in the field of food and nutrition. Extracting protein concentrate from pumpkin leaves as a by-product and incorporating it into nanofibers provided a homogeneous nanofiber structure and a sustainable and plant-derived carrier. This combination of gelatin and leaf protein concentrates in nanofibers delivered a biocompatible matrix with a promising potential for vitamin B12 delivery in the food industry. The obtained nanofibers ensured a slower release of vitamin B12 over time while sustaining thermal stability. This approach has several potential benefits, such as the prolonged shelf life of fortified foods and the development of functional food products with improved nutritional profiles. Additionally, using plant-based proteins from some crops aligns with the growing demand for sustainable and eco-friendly food technologies.

## Figures and Tables

**Table 1:** Viscosity, surface tension, density, and conductivity of the leaf protein concentrate (LPC) solution, gelatin solution (Ge), solution of vitamin B12 in 30% acetic acid (B12 solution), gelatin with the leaf protein concentrate solution (Ge+LPC), gelatin with vitamin B12 solution (Ge+B12) and gelatin with the leaf protein concentrate and vitamin B12 solution (Ge+LPC+B12).

Sample	Viscosity (mPa s)	Surface Tension (mN/m)	Density (g/mL)	Conductivity (mS/cm)
LPC	1.15 ± 0.08 ^a^	33.4 ± 0.06 ^c^	0.999 ± 0.000 ^a^	2.76 ± 0.01 ^b^
Ge	35.00 ± 0.04 ^c^	33.5 ± 0.08 ^c^	1.082 ± 0.000 ^d^	4.10 ± 0.00 ^d^
B12 solution *	1.20 ± 0.04 ^a^	35.5 ± 0.02 ^e^	1.037 ± 0.000 ^b^	1.81 ± 0.07 ^a^
Ge+LPC	40.00 ± 0.02 ^e^	30.9 ± 0.01 ^b^	1.085 ± 0.000 ^e^	4.51 ± 0.02 ^e^
Ge+B12	36.63 ± 0.05 ^d^	29.1 ± 0.00 ^a^	1.069 ± 0.001 ^c^	3.83 ± 0.15 ^c^
Ge+LPC+B12	30.17 ± 0.03 ^b^	34.4 ± 0.47 ^d^	1.070 ± 0.000 ^c^	5.46 ± 0.07 ^f^

* in 30% acetic acid, different letters within the same column indicate statistically significant differences (*p* < 0.05).

**Table 2:** The differential scanning calorimetry data for the leaf protein concentrate (LPC), gelatin powder (Ge powder), gelatin nanofibers (Ge), gelatin with the leaf protein nanofibers (Ge+LPC), Gelatin with vitamin B12 nanofibers (Ge+B12), and gelatin with the leaf protein concentrate and vitamin B12 nanofibers (Ge+LPC+B12).

Sample	Temperature			ΔH (J/g)
	Onset	Peak	Offset	
LPC	58.79	96.11	124.71	161.65
GE powder	76.25	118.24	168.86	251.32
Ge	67.75	90.24	161.72	490.02
Ge+LPC	66.38	95.53	130.93	310.53
Ge+B12	33.81	57.39	88.12	138.58
Ge+LPC+B12	63.33	89.99	124.32	232.39

**Table 3:** Water activity values of the leaf protein concentrate (LPC), gelatin powder (Ge powder), gelatin nanofibers (Ge), gelatin with the leaf protein nanofibers (Ge+LPC), gelatin with vitamin B12 nanofibers (Ge+B12), and gelatin with the leaf protein concentrate and vitamin B12 nanofibers (Ge+LPC+B12).

Sample	Aw
LPC	0.393 ± 0.031 ^a^
Ge powder	0.392 ± 0.042 ^a^
Ge	0.336 ± 0.041 ^a^
Ge+LPC	0.341 ± 0.001 ^a^
Ge+B12	0.361 ± 0.001 ^a^
Ge+LPC+B12	0.376 ± 0.033 ^a^

Different letters within the same column indicate statistically significant differences (*p* < 0.05).

**Table 4:** Model parameters of vitamin B12 release from gelatin-LPC-fortified fibers (Ge+LPC+B12) into aqueous medium.

Model	Parameter	Ge+LPC+B12
Higuchi	k	15.44
	R^2^	0.935
	RMSE	5.476
Ritger-Peppas	k	20.93
	n	0.389
	R^2^	0.947
	RMSE	4.943
Kopcha	A	19.32
	B	−0.877
	R^2^	0.943
	RMSE	5.101
Pepas–Sahlin	k_1_	20.156
	k_2_	1.055
	m	0.354
	R^2^	0.944
	RMSE	5.09

## Data Availability

The original contributions presented in the study are included in the article, further inquiries can be directed to the corresponding author.
